# Methods for Overcoming Chemoresistance in Head and Neck Squamous Cell Carcinoma: Keeping the Focus on Cancer Stem Cells, a Systematic Review

**DOI:** 10.3390/cancers16173004

**Published:** 2024-08-29

**Authors:** Maria Eleonora Bizzoca, Vito Carlo Alberto Caponio, Lorenzo Lo Muzio, Pier Paolo Claudio, Antonio Cortese

**Affiliations:** 1 Department of Clinical and Experimental Medicine, University of Foggia, 71122 Foggia, Italy; mariaeleonora.bizzoca@unifg.it (M.E.B.); vitocarlo.caponio@unifg.it (V.C.A.C.); lorenzo.lomuzio@unifg.it (L.L.M.); 2 Department of Pharmacology and Toxicology, Cancer Center & Research Institute, University of Mississippi Medical Center, Jackson, MS 39216, USA; 3 Unit of Maxillofacial Surgery, Department of Medicine, Surgery, and Dentistry, University of Salerno, 84084 Salerno, Italy

**Keywords:** oral squamous cells carcinoma, head and neck squamous cell carcinoma, cancer stem cells, ChemoID, functional precision medicine, natural products

## Abstract

**Simple Summary:**

Head and Neck Cancer is often diagnosed at an advanced stage, resulting in poor long-term prognosis. Additionally, this cancer is resistant to chemotherapy. This systematic review article aims to examine the literature on published methods to overcome resistance to chemotherapy for head and neck cancer.

**Abstract:**

According to the “cancer stem cell” (CSCs) theory, tumors are a diverse and expanding group of malignant cells that originate from a small number of CSCs. Despite treatment, these cells can still become active and proliferate, which can result in distant metastasis and local recurrences. A new paradigm in cancer treatment involves targeting both CSCs and the cancer cells in a tumor. This review aims to examine the literature on methods published to overcome chemoresistance due to the presence of CSCs in head and neck cancers. The review was registered with PROSPERO (ID# CRD42024512809). After Pub Med, Scopus, and WoS database searches, 31 relevant articles on oral squamous cell carcinoma (OSCC) were selected. Compounds that increased chemosensitivity by targeting CSCs in head and neck squamous cell carcinoma (HNSCC) were divided into (1) natural products, (2) adjuvant molecules to traditional chemotherapy, and (3) CSCs targeting patient-specific fresh biopsies for functional precision medicine.

## 1. Introduction

According to the “cancer stem cell” (CSCs) theory, tumors are a diverse and expanding group of malignant cells that originate from a small number of CSCs [1,2,3,4]. Despite treatment, these cells can still become active and proliferate, which can result in distant metastasis and local recurrences [5].

Head and neck cancer (HNC) is a broad range of diseases originating in the head and neck region, that include neoplasms from the oral cavity, nasopharynx, oropharynx, hypopharynx, and larynx [6]. Different epidemiology, etiology, and therapeutic strategies can be linked to the disease group as a whole. Given the complexity of head and neck cancer, diagnostic-therapeutic choices must be made by multidisciplinary teams with training not only in the specific treatment but also in supportive care. In 2019, 53,000 new cases and 10,860 deaths related to HNC were observed in the U.S. [7]. In 2030, there are projected to be 439,000 mouth and oropharynx cancer cases, according to estimates from the World Health Organization [8].

Surgery, radiotherapy, chemotherapy, targeted therapy, and immunotherapy are standard-of-care treatments for HNC, generally administered in combination [9]. In most cases, the disease is diagnosed at an advanced stage and for this reason, there is no good long-term prognosis [10]. Head and neck squamous cell carcinoma (HNSCC), a major histological type of HNC, is the sixth most common cancer worldwide and the most frequent form of malignancy found in the oral cavity [11,12]. The standard medical regimen for treating oral squamous cell carcinoma (OSCC) often consists of one or more conventional therapies, such as radiation, surgery, and chemotherapy such as docetaxel, 5-fluorouracil, or cisplatin (CDDP) [13]. The current treatment protocol for patients with locally advanced HNSCC is concomitant platinum-based chemo-radiotherapy (CRT) or surgery followed by adjuvant radiation or chemoradiotherapy. The response rate for patients with recurrent and/or metastatic HNSCC is 30–40%, and the median survival is 6–9 months, with platinum-based chemotherapy and 5-fluorouracil (5-FU) being the treatment option. Patients who are resistant to platinum have few options and a very poor chance of survival with second-line therapies [14]. Due to the difficulties in treating head and neck malignant neoplasms, investigations of novel strategies and therapies are required to find more effective treatments for these malignant tumors. Over the years, numerous attempts have been made to develop an ex vivo anti-cancer test that could aid in identifying the best treatment options for each patient while minimizing toxicities.

It has been demonstrated through animal xenograft models that only a subset of cancer cells within each tumor can initiate tumor growth. The “cancer stem cells” (CSCs) subset is the operational definition of this pool of cancer cells. According to the “cancer stem cell” theory, tumors are a diverse and expanding group of malignant cells that originate from a small number of CSCs [1,2,3,4]. Although CSCs form a very small proportion of the tumor cell population, they play a significant role in determining outcomes. Generally, CSCs refer to the cancer cells capable of self-renewal and differentiation, which makes them resistant to radiotherapy and chemotherapy; conventional therapy may not be effective for them if they remain inactive for long periods. Despite treatment, these cells can still become active and proliferate, which can result in distant metastasis and local recurrences [5]. Like normal stem cells (NSCs), CSCs exhibit stem cell characteristics that include self-renewal, long life span, migration capacity, and drug resistance [15]. In HNSCC, the CSCs are essential to the initiation, metastasis, and progression of the disease, as well as its treatment resistance [16]. The CSCs proliferate slowly and can differentiate into phenotypically heterogeneous and aberrant progeny [4,17].

HNSCC’s survival rate and prognosis have not significantly improved over the years, even despite advances in treatment [18]. A new paradigm in cancer treatment involves targeting both CSCs and the cancer cells in a tumor. CSCs are thought to arise either from transformed adult stem cells or through the dedifferentiation of somatic cells [19]. The behavior of CSCs is influenced by various transcription factors that mediate pluripotency and self-renewal, such as c-MYC, Nanog, OCT-3/4, SOX2, and KLF4. Additionally, stemness-related signaling pathways, including Wnt/β-catenin, Hedgehog, Notch, JAK/STAT, TGF-β/SMAD, PI3K/Akt, and NF-kB, play a role [20]. This is further modulated by communication within the tumor microenvironment (TME) through intercellular and extracellular matrix (ECM) interactions [20]. The identification of CSCs in solid tumors involves the detection of specific markers either on the cell surface (such as CD10, CD24, CD44, CD90, CD133, CD271, EpCAM, LGR5) or within the cell (ALDH1, Nanog, OCT3/4, BMI-1, SOX2) [21]. HNSCC cells within the CSC population are characterized by elevated expression of specific surface proteins (such as CD44 and CD133), pluripotency-related transcription factors (SOX2, OCT4, and Nanog), and heightened aldehyde dehydrogenase (ALDH) activity [22].

A correlation between epithelial-mesenchymal transition (EMT) and CSC features, which signify the ability of cancer cells to reversibly flip to a different phenotype to adapt to the changing tumor microenvironment, has been suggested by recent studies on a variety of carcinomas [23,24]. CSCs in OSCC exhibit remarkable phenotypic plasticity, allowing them to transition between two phenotypes: both CD44^high^ ESA^high^ epithelial and proliferative (non-EMT CSCs) and CD44^high^ ESA^low^ mesenchymal and migratory (EMT CSCs), where ESA is an epithelial marker (also known as epithelial-specific antigen or EpCAM) [25]. The occurrence of hybrid epithelial/mesenchymal (E/M) states between the two extremes of pure epithelial or mesenchymal phenotypes must also be considered [26].

Several studies have been carried out on the subject, but it should be noted that different chemosensitivity resulted between tumors of the same histological type from ex-ante studies [17,27,28,29,30,31]. In these studies, different chemosensitivity among patients with the same disease (oral squamous cell carcinoma, OSCC) was analyzed in a very specific area (oral cavity), for a specific histotype (SCC).

Precision medicine is being deployed to support oncologists during the therapy decision-making process of several types of tumors. Research and clinics have at their disposal another important diagnostic tool, the CHEMOID^®^ assay. By this method, very precise and more sensitive results can be achieved thanks to *ex-ante-patient specific* analysis, compared to current guidelines based on post-treatment of patient pools (*ex-post*) in clinical trials. ChemoID is a functional precision medicine assay based on living cancer cells cultured from fresh patient tumor biopsies. The assay directly measures the effects of a panel of chemotherapies and their combination on CSCs and the bulk of the tumor cells [27,30,31]. Although this assay is an in vitro method, the outcomes observed following guided therapy are more effective than the empirical choice of chemotherapies as demonstrated in a randomized clinical trial and several other clinical studies [27,28,29,30,31,32,33,34,35].

This technology platform in the future could lead to individualized chemotherapy based on the patient’s specific tumor characteristics for several types of cancers.

This review aims to examine the literature on methods published in both preclinical and clinical studies to overcome chemoresistance due to the presence of CSCs in head and neck cancers.

## 2. Materials and Methods

The research design employed was a systematic review of the literature, using a specific search strategy as described below. This review includes all the original articles that investigate diagnostics aimed at overcoming chemoresistance in head and neck tumors. Studies published in the English language and those published from 2001 onward were included in this review. The review was registered with PROSPERO (ID# CRD42024512809), link: https://www.crd.york.ac.uk/PROSPERO/display_record.php?RecordID=512809 (accessed on 26 February 2024).

In our search, we considered randomized controlled trials (RCTs), retrospective studies, case-control studies, and qualitative research. However, we excluded opinion pieces, articles, magazines, newspapers, commentaries, editorials, and systematic reviews.

For our search strategy, PRISMA guidelines were followed, and the search dates were from 21 November 2023, to 20 March 2024. The search strategy was entered in PubMed, Scopus, and Web of Science by a combination of specific keywords and Boolean operators, like AND-OR.

The MeSH terms for the search were: (neoplastic stem cells), (drug resistance, neoplasm), (head and neck neoplasms). The search terms used for PubMed were: ((CSC OR “neoplastic stem cells” OR “cancer stem cells” OR “tumor-initiating cells”) AND (“drug resistance, neoplasm” OR chemoresistance OR “chemotherapy resistance” OR “treatment failure”) AND (HNSCC OR “head and neck neoplasms” OR “mouth neoplasms” OR OSCC OR “head and neck squamous cell carcinoma” OR “oral squamous cell carcinoma” OR “oral cancer”)). The search terms used for Scopus were: ((csc OR “neoplastic stem cells” OR “cancer stem cells” OR “tumor-initiating cells”) AND (“drug resistance, neoplasm” OR chemoresistance OR “chemotherapy resistance” OR “treatment failure”) AND (hnscc OR “head and neck neoplasms” OR “mouth neoplasms” OR oscc OR “head and neck squamous cell carcinoma” OR “oral squamous cell carcinoma” OR “oral cancer”)). The search terms used for Web of Science were: ((CSC OR “neoplastic stem cells” OR “cancer stem cells” OR “tumor-initiating cells”) AND (“drug resistance, neoplasm” OR chemoresistance OR “chemotherapy resistance” OR “treatment failure”) AND (HNSCC OR “head and neck neoplasms” OR “mouth neoplasms” OR OSCC OR “head and neck squamous cell carcinoma” OR “oral squamous cell carcinoma” OR “oral cancer”)).

**Figure 1:**
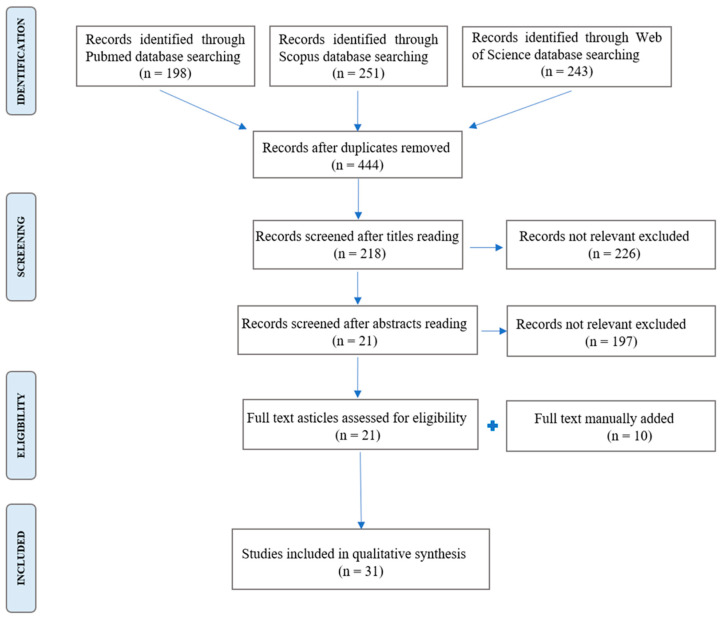
Flowchart of the selection process.

From the PubMed database search, we obtained 198 results; from Scopus, we obtained 251 results; from Web of Science, we obtained 243 results. By merging all the results in EndNote X9 (692 papers), 248 article duplicates were automatically deleted, resulting in a total of 444 studies. After reading the titles of all 444 articles, 226 papers not related to the research topic were deleted. Subsequently, the abstracts of the remaining 218 papers were screened and 197 were eliminated because their topic was not related. Finally, we restricted the search to 21 published papers that were relevant to the topic of the study. To these were added manually 10 studies based on the rationale and methods analysis of relevant studies in the field as detected by the authors. In total, 31 relevant studies have been included in this systematic review (Figure 1).

The inclusion criteria of this study were: (a) in vitro studies, studies in humans, and studies in animals; (b) studies in the English language; (c) studies that analyze molecules that fight chemoresistance of CSCs in the head and neck district. The exclusion criteria of this study were: (a) studies not in English; (b) review; (c) studies that analyze any other tumor than head and neck cancer; (d) studies that show an aggressor pathway without defining the molecules or the tools usable.

Two reviewers (M.E.B and V.C.A.C.) autonomously collected data from the 31 papers included in the systematic review regarding key outcomes related to the study questions using a data extraction sheet. In instances where disparities arose between the two reviewers, a third reviewer (L.L.M.) was consulted. In such cases, the extracted data underwent reassessment or resolution through discussion. As this is a systematic review involving no direct human interaction or primary data collection, and instead, focuses on the examination of the previously documented literature, there were no significant ethical concerns.

## 3. Results

From the analysis of the studies included in this systematic review, we found the results described below. The majority of the studies we found during our systematic review of the literature were conducted on established cancer cell lines derived from oral cancer that were cultured for several passages and that are described in Section 3.1 and Section 3.2. Unfortunately, these studies do not take into consideration the phenotypic differences between HPV-positive and HPV-negative cancers and the gender-based differences in treatment response, and for these reasons, we will not discuss them in this review.

**COMPOUNDS THAT INCREASE CHEMOSENSITIVITY BY TARGETING CANCER STEM CELLS IN HNSCC**

### 3.1. Natural Products

Patients with OSCC may experience both acute and long-term adverse effects from conventional treatments that have been utilized more extensively up to this point, not just CDDP but also radiotherapy. The most common side effects following radiation therapy have been trismus, dry mouth, oral mucositis, caries, and osteoradionecrosis [36]. Conventional chemotherapy can also have several negative effects, including anemia, vomiting, nephrotoxicity, hepatotoxicity, and neurotoxicity, as well as infections and oral irritation [37]. Furthermore, these therapies may still cause issues with swallowing, deformity, excessive medical expenses, and impairment of several vital processes, including breathing, speech, smell, taste, and mastication. Apart from the unfavorable consequences of conventional treatment alternatives for oral cancer, the prognosis and survival rates are still low or nonexistent despite advancements in therapeutic research [38]. Developing novel treatments targeted at eliminating CSCs and blocking epithelial-mesenchymal transition (EMT) becomes essential for treating OSCC effectively, given the significant role these molecular mechanisms play in the development of malignancy. These treatments should not only target the tumor mass but also the remaining cell cluster that is closely linked to metastasis, recurrence, and resistance to standard therapies [23,24].

Herbal medications have been extensively explored in tumor cells today to create techniques to minimize adverse effects for individuals undergoing treatment and provide alternatives to compose adjuvant or combined treatments for cancer therapy [39].

Table 1 summarizes the mechanisms of action on CSCs of natural products and their adjuvant effects on CSCs.

The *Ganoderma lucidum* (GL) *Karst*, mushroom, also known as the “plant of immortality”, is traditionally used in Eastern countries’ medicine to improve the vitality of the human body, assist in longevity, and the treatment of various human diseases, including cancer [40]. Numerous anti-tumor characteristics of GL have been linked to particular elements found in this mushroom, including glycoproteins, water-soluble heteropolysaccharides, and polysaccharides, particularly β-glucans, whose pharmacological actions primarily entail preventing the growth of tumor cells or causing them to die [41]. On the other hand, not much is known about how Ganoderma lucidum polysaccharides (GLPS) affect CSCs, particularly in cases of oral squamous cell carcinoma (OSCC). This is particularly true for tongue squamous cell carcinoma (TLSCC), the most prevalent and poorly treated subtype of OSCC due to its potential for glossectomy and other functional and cosmetic consequences [38]. De Camargo et al. sought to evaluate the impact of GLPS on the aggressive potential of OSCC in vitro, concentrating on the characteristics and roles of CSCs, the EMT phenotype, and molecular pathways associated with drug susceptibility [13]. In this study, GLPS-treated cells (commercially available tongue cancer cell line SCC-9, ATCC-CRL-1629) showed a dose-dependent decrease in CSC colony formation, when compared to the control group. Additionally, following GLPS therapy, the tumor cells did not exhibit the sphere-forming ability that is indicative of CSCs, hence indicating that GLPS might work in tandem to induce the epithelial CSC phenotype and ultimately aid in the development of a phenotype that is more sensitive to chemotherapy [13]. Biological evidence for the application of polysaccharides from the millennium compound *Ganoderma lucidum* to contemporary targeted therapy is shown in this study’s conclusion, which may increase the effectiveness of current treatments for oral cancers, particularly tongue squamous cell carcinoma. Within this framework, GLPS may serve as viable therapeutic co-adjuvants, augmenting CSC elimination, impeding invasion and metastasis, increasing medication sensitivity, and potentially ameliorating the patient’s quality of life [13]. *Ganoderma lucidum*, which is also known as Lingzhi, is a medicinal mushroom that has been used for millennia as an herbal treatment in traditional medicine. Lingzhi species have been shown to have medicinal qualities, including immunomodulation, anti-cancer, and anti-inflammatory effects [42,43,44]. It contains a range of bioactive compounds, including polysaccharides, fungal immunomodulatory proteins, and triterpenoids (FIPs) [44]. A study conducted by Wang et al. [45] assessed the effect of a FIP from Ganoderma microsporumon (GMI) on the stemness features and CDDP resistance in oral cancer stem cells (OCSCs). This study showed that GMI’s tumor-suppressive action was achieved by blocking IL-6/Stat3 signaling. Numerous investigations have demonstrated that Stat3 inhibition increases the effectiveness of chemotherapeutic drugs in OSCC [46,47] and that Stat3 hyperactivation is linked to tumor growth and treatment resistance [48,49].

Furthermore, it has been shown that the primary autocrine/paracrine factor for Stat3 activation in OSCC is IL-6 [50]. Targeting IL-6/STAT3 signaling has been demonstrated in numerous studies to decrease CSCs both in vitro and in vivo [51,52]. The findings of Wang et al. analysis revealed GMI’s anti-OCSC action both in vitro and in vivo. Furthermore, GMI’s tumor-suppressive action was achieved by controlling the IL-6/Stat3 pathway. GMI also had chemosensitizing properties and might be used as an adjuvant to CDDP to lessen the risk of cancer recurrence. This study shed fresh light on the application of GMI as an anti-tumor drug in the treatment of OSCC.

*Sulforaphane* (SF), a phytochemical found in large quantities in cruciferous plants, has demonstrated promising anti-inflammatory, antioxidant, and antitumor effects [53]. Recent studies have suggested that SF exerts its antitumor effects by inhibiting both cell proliferation and cell cycle mechanisms, promoting apoptosis, and protecting precancerous cells from methylation [53,54]. However, its effect on CSCs in HNSCC, whether alone or in combination with conventional chemotherapy, remains poorly understood. Elkashty et al. investigated whether SF could be a potent agent to enhance the effectiveness of CDDP and 5-fluorouracil (5-FU) chemotherapy on HNSCC stem cells from commercially available laryngeal cancer cell line SCC-12 (ThermoFisher #18292011) and tonsillar cancer cell line SCC-38 (Cellosaurus UM-SCC38, tonsillar SCC, RRID: CVCL_7749), by determining its mechanisms of action [55]. Potential mechanisms of action included the stimulation of caspase-dependent apoptotic pathway, inhibition of SHH pathway and decreased expression of SOX2 and OCT4. This study’s novel discovery was that SF might be applied in conjunction with CDDP and 5-FU to increase their toxicity against the more resilient CSCs in HNSCC. In particular, at lower treatment doses, adding 3.50 µM of SF substantially doubled the efficacy of CDDP and amplified the effect of 5-FU by ten. Serum concentrations of 3.50 µM SF in the human body are achievable clinically by eating fresh broccoli sprouts. It has been found that the plasma concentration of SF rises to 2.50 µM/L in about three hours after consuming 40 g of broccoli [56]. Additionally, supplementing SF during conventional CDDP or 5-FU chemotherapy treatments prevented the potential of CSCs to proliferate. In summary, these findings imply that SF improved the anticancer effects of chemotherapy both in vitro and in vivo, enabling a dose reduction of the chemotherapy drugs. In the clinical context, SF in combination with conventional chemotherapy (CDDP and 5-FU) may offer a more effective treatment strategy.

A biphenyl-type neolignan called *Honokiol* is taken from the bark of the Magnolia plant, which is used in traditional Chinese herbal medicine [57]. This bioactive molecule has several pharmacological properties, including antioxidant, anti-inflammatory, neurotrophic, and anti-cancer effects [57]. Honokiol has been demonstrated to suppress CSCs and decrease drug resistance in glioblastoma cells, and it has demonstrated an antiproliferative effect on OSCC through the modulation of specificity protein 1 [58]. Honokiol was also found to reduce sphere formation and xenograft tumor growth of oral CSCs (OCSCs) in one of the most recent investigations [59]. Consequently, to clarify the effectiveness of using honokiol as an adjuvant agent in the eradication of OSCC, Chang et al. investigated cancer stemness, cell motility, and chemoresistance of OCSCs from commercially available tongue cancer cells SAS (Cellosaurus SAS, CVCL_1675), gingival cancer cells OECM-1 (Cellosaurus OECM-1, CVCL_6782), gingival cancer lymph nodal metastasis cells GNM (Cellosaurus GNM, WL58) and normal gingival epithelial S-G cells (F.H. Kasten, East Tennessee State University, Quillen College of Medicine, Johnson City, TN, USA) following treatments with honokiol [60] and demonstrated that it was an appropriate adjuvant drug to lower CD44 expression and ALDH1 activity in CSCs, which in turn inhibited the cells’ capacity to proliferate and become malignant. It has been proven that ALDH1 expression is closely connected with tumor differentiation [61]. In this study [60], the authors demonstrated that honokiol suppressed the phosphorylation of STAT3, maybe through the decrease of IL-6, a growth factor that stimulated STAT3. It has been suggested that IL-6/STAT3 signaling is essential for the survival of CSCs in oral cancer [62]. In line with the existing evidence, Chang et al. demonstrated that honokiol may increase the effectiveness of CDDP and amplify the side effects of chemotherapy [60]. Furthermore, it has been demonstrated that 5-fluorouracil and honokiol together have an additive effect on OSCC by apoptotic induction [63].

The *phenanthrene alkaloid berberine* was extracted from the bark and roots of plants belonging to the Berberis genus (Berberidaceae family). Its many biological actions, such as anti-microbial, anti-inflammatory, and antioxidant qualities, have led to its traditional usage in Oriental medicine [64,65]. Its anti-tumor properties, which include anti-metastasis, apoptosis and autophagy activation, and suppression of the epithelial-to-mesenchymal transition (EMT) in a variety of tumor cell types, have been the subject of recent investigations [66,67]. Additionally, several studies have shown that it causes oral cancer cells to undergo apoptosis [68,69]. Lin et al. [68] assessed the impact of berberine on OSCC-CSCs population from SAS and OECM-1 cell lines compared to normal gingival S-G cells. The following parameters were evaluated: (1) invasion capability in vitro, (2) self-renewal, (3) colony formation, (4) ALDH1 activity, and (5) tumorigenicity in vivo. Furthermore, the authors discovered that: berberine administration increased the tumor’s sensitivity to chemotherapy and berberine supplementation reduced miR-21 expression in a dose-dependent manner. Additionally, CSC characteristics such as self-renewal, migration, invasion potential, and CSC marker expression were decreased by miR-21 suppression. These findings suggest that miR-21 may be essential for berberine’s anti-CSC activity. In summary, the authors showed that berberine inhibits tumor growth and increases chemosensitization in OSCC-CSCs by controlling miR-21.

A licorice flavonoid called *Isoliquiritigenin* (ISL) has been shown to have a variety of medicinal uses, including analgesic, anti-inflammatory, spasmogenic, and spasmolytic qualities [70,71]. ISL has been shown to impede tongue squamous carcinoma cells in HNSCC through the antioxidant mechanism [72]. Hu et al. [73] in this study on CSCs (compared SAS and OECM-1 cell lines), and investigated how ISL affected OSCC-CSCs’ ability to self-renew, spread, and become more sensitive to chemotherapy. The authors discovered that ISL inhibited the expression of markers for CSCs, as well as their capacity for self-renewal and colony formation through the downregulation of GRP78. Additionally, these results indicated that ISL improved the therapeutic impact of CDDP with decreased ABCG2 expression and CSC characteristics. Ultimately, these findings indicate that ISL possesses the ability to function as an anti-CSC drug by inhibiting many CSC traits, including the expression of CSC markers, self-renewal, metastasis, colony formation, and chemotherapy enhancement; a key factor in these events was the GRP78 in OSCC-CSCs being reduced via ISL. All in all, these results provide credence to the possibility of using ISL in clinical settings as a nutritional intervention in addition to chemotherapy.

*Heat-shock proteins* (Hsps) are mostly triggered by environmental stressors and serve as molecular chaperones. Hsps are divided into two groups: low-molecular-weight Hsps (like Hsp27) and high-molecular-weight Hsps (like Hsp90 and Hsp70) [74]. Apart from its typical role as a chaperone, Hsp27 has been observed to be overexpressed in tumors of the breast, ovary, and head and neck regions [75]. Furthermore, it has been shown that Hsp27 is linked to the generation of cancer cells with stem-cell-like characteristics and chemoresistance in breast cancer and numerous other malignancies [74]. There are, however, few findings on Hsp27’s role in OSCC prognosis and medication resistance. The main flavonoid (3,30,40,5,7-penta-hydroxy-flavanone) that is frequently derived from cranberries, blueberries, apples, and onions is *Quercetin (Qu).* It has a broad range of bio-pharmacological characteristics, and because of its potent antioxidant and free-radical-scavenging abilities, it may present interesting new possibilities for the creation of more successful chemopreventive and chemotherapeutic approaches [76]. A study by Chen et al. [74] on SCC-25 tongue cancer cells (ATCC CRL-1628), sheds light on the mechanism underlying OSCC treatment resistance. An important factor in CSC-mediated CDDP resistance in oral cancer is the p38 MAPK–Hsp27 axis. Combining Qu with conventional chemotherapy to target this axis may be a therapeutic approach to help patients with OSCC have a better prognosis [74].

*Anisomeles Indica* (L.) Kuntze, a Chinese traditional herbal medicine, yields *Ovatodiolide* (Ova), a bioactive macrocyclic diterpenoid molecule with demonstrated anti-inflammatory, anti-angiogenesis, antibacterial, antiviral, antioxidant, and anticancer properties [77]. In a study by Lin et al. [77], the survival and multiplication of oral CSCs derived from the following cancer cell lines: SAS, FaDu (ATCC-HTB-43), TW2.6 (Cellosaurus TW2.6, CVCL_GZ05), and HSC-3 (Millipore Sigma SSC-193) are markedly inhibited by Ova, either alone or in conjunction with CDDP. Ova also suppresses the development of oral spheres, decreases clonogenicity, increases CDDP sensitivity, and dysregulates the JAK2/STAT3 signaling pathway in addition to its anti-CSC effects [77]. A framework for further investigation into Ova’s potential therapeutic role as a novel small molecular inhibitor of STAT3, which is constitutively activated in most malignancies, including oral cancer, has been established by the replication of its anticancer and CDDP-enhancing activities in preclinical in vivo oral cancer models [77].

A kinetochore protein called spindle pole body component 25 (SPC25) was found to be overexpressed in CDDP-resistant (CR) cells derived from SAS and CGHNC-8 cell lines chronically exposed to CDDP, and had a strong negative correlation with the survival of HNC patients [78]. CGHNC-8 is a cancer cell line established from an oral cancer squamous cell carcinoma of a patient from Chang Gung Memorial Hospital, Taoyuan, in Taiwan. In HNC cells, silencing this molecule reduced CR and inhibited cancer stemness. Chen et al. [78] demonstrated that a natural extract component called *Celastrol* showed strong cytotoxic effect in CR cells and was shown to be an SPC25 inhibitor. The creation of SPC25 inhibitors, such as celastrol, may offer a fresh method for sensitizing CDDP in the treatment of HNC that is resistant to it.

In a study by Jiang et al. [79], it is affirmed that nuclear receptors such as retinoid X receptor-α (RXRα) are targets for cancer prevention and treatment across a range of cancer types, but the mechanism of how RXRα regulates CSCs remains unknown. Jiang et al. discovered that the enriched HNSCC CSCs from FaDu cells and the tissues from head and neck squamous cell cancer (HNSCC) have elevated levels of RXRα [79]. In HNSCC cells, overexpression of RXRα was able to enhance the CSC-like characteristics, while RXRα knockdown was able to suppress the stemness. In HNSCC cells, on the other hand, low doses of cisplatin (CDDP) enhanced the CSC-like characteristics and RXRα expression. Additionally, CDDP-induced CSCs were significantly influenced by the Wnt signaling pathway. Furthermore, in a mouse xenograft model, *Curcumin*, a plant polyphenol, which is an effective anticancer compound, decreased the expression of stemness markers and reversed the CD44 + and side population (SP) cell ratios that CDDP had induced by inhibiting RXRα. When combined with CDDP, curcumin inhibited tumor development even further [79].

The bioactive polyphenolic compound known as *Magnoliol* (5,5′-diallyl-2,2′-dihydroxybiphenyl) is extracted from the root and stem bark of Magnolia officinalis. Numerous biological attributes have been discovered in it, including anti-oxidant, anti-inflammatory, and anti-cancer, and anti-inflammation effects [80,81]. It has been demonstrated that *magnoliol* can limit the growth of tumors and the spread of different kinds of malignancies [82]. It has recently been shown that the chemopreventive actions of magnolia extract against oral cancer are achieved by suppressing mitochondrial respiration at complex I of the electron transport chain [83]. A different study revealed that magnolia extract has anti-oral cancer proliferation properties and worked well as an in vivo cancer chemopreventive dietary supplement [84]. Peng et al. demonstrated that magnolol could be an advantageous dietary supplement for OSCC patients since it inhibited the growth of cancer cells and decreased the capacity of OSCC-CSCs from SAS, OECM1, and GNM cell lines, to self-renew and spread through the IL-6/Stat3 pathway [85].

The pineal gland is the primary producer of the hormone *melatonin*, also known as N-acetyl-5-methoxytryptamine, which plays a significant role in many biological processes including immunity, sleep, circadian rhythm, and bone formation [86]. Melatonin is thought to protect normal cells by modifying mitochondrial function and reducing apoptotic signaling [87]. On the other hand, melatonin increases the death caused by anti-cancer drugs in several human cancer cell lines [88]. This suggests that melatonin sensitizes cancer cells to chemotherapy-induced apoptosis by causing malfunction in the mitochondria [88]. However, the precise impact of melatonin on anti-cancer drug-induced apoptosis in OSCCs remains unclear. Shigeishi et al. [89] demonstrated that melatonin is essential for enhancing the cytotoxicity that CDDP induces in CD44^high^ OSCC cells from the oral cavity OM-1 cell line (Cellosaurus OM-1, CVCL_W941). Additionally, in mesenchymal-like CD44^high^ OSCC cells, DERL1 contributes to resistance to CDDP-induced cell death. Through DERL1 attenuation, melatonin-induced miR-181c-5p amplifies CDDP-induced cell death [89]. Consequently, melatonin may contribute to the cytotoxicity caused by chemotherapy in CD44^high^ OSCC cells that exhibit mesenchymal traits [89]. The study’s findings imply that, in CD44^high^ OSCC cells, melatonin and DERL1 have a significant relationship: Melatonin sensitized CD44^high^ cells to CDDP-induced cell death [89].

**Table 1:** Natural products adjuvant to chemotherapy and their effects on CSCs.

	Mechanism of Action on CSCs	Effect on Conventional Chemotherapy
**Ganoderma lucidum (GL)** [52,53]	Fungal immunomodulatory proteins block IL-6/Stat3 signaling.	Decreased resistance to CDDP.
**Sulforaphane (SF)** [55,56]	Stimulation of caspase-dependent apoptotic pathway, inhibition of Sonic Hedgehog (SHH) pathway and decreased expression of SOX2 and OCT4.	Enhances the effectiveness of CDDP and 5-fluorouracil (5-FU) chemotherapy.
**Honokiol** [61,62,63,64]	Lowers CD44 expression and ALDH1 activity in CSCs; Suppresses the phosphorylation of STAT3, possibly through the decrease of IL-6, a growth factor that stimulates STAT3.	Increases effects of CDDP, and it has an additive effect on 5-fluorouracil.
**Phenanthrene alkaloid Berberine** [69]	Reduces miR-21 expression in a dose-dependent manner; decreases CSCs marker expression.	Increases effects of CDDP.
**Isoliquiritigenin (ISL)** [73,74]	Downregulates GRP78 and decreases CSCs marker expression; Decreases ABCG2 expression.	Increases effects of CDDP.
**Quercetin (Qu)** [75]	Downregulates HSP27; Disrupts the p38 MAPK-Hsp27 axis.	Increases effects of CDDP.
**Ovatodiolide (Ova)** [78]	Dysregulates the JAK2/STAT3 signaling pathway.	Increases effects of CDDP.
**Celastrol** [79]	Spindle Pole body Component 25 (SPC25) inhibitor.	Increases effects of CDDP.
**Curcumin** [80]	Decreases the expression of stemness markers and reverses the CD44 + and side population (SP) cell ratios by inhibiting RXRα.	Increases effects of CDDP.
**Magnoliol** [84,85,86]	Suppresses mitochondrial respiration at complex I of the electron transport chain; inhibits the IL-6/Stat3 pathway.	Increases effects of CDDP.
**Melatonin (N-acetyl-5-methoxytryptamine)** [88,89]	Upregulates *miR*-*181c*-*5p* expression, which decreases DERL-1 expression.	Enhances cisplatin-induced cell death in mesenchymal-like CD44^high^ cells.

### 3.2. Adjuvant Molecules to Traditional Chemotherapy

Several molecules have been studied on head and neck cancer cells to increase the effects of traditional chemotherapies to provide adjuvant and improved treatments for cancer therapy. Table 2 summarizes the mechanisms of action on CSCs of synthetic molecules and other molecules and their adjuvant effects on CSCs.

In a study conducted by Praharaj et al. [90], the efficacy of ML-385 (a pharmacological inhibitor of NRF2) and chloroquine (CQ) on FaDu and tongue carcinoma Cal-33 cells (DSMZ, ACC447) was evaluated. When compared to either CQ + CDDP or ML-385 + CDDP treatment, it was found that the combined treatment (CQ + ML-385 + CDDP) effectively reduces CD44 staining (representing CD44+ populations). Additionally, targeted inhibition with a genetic (siNRF2) or pharmacological (ML-385) inhibitor significantly reduces cancer stemness in oral cancer. The combination of CQ and ML-385, according to the authors, greatly enhances the therapeutic efficacy of CDDP in causing the targeted eradication of oral CD44+ CSCs. Consequently, to determine if this strategy is useful in treating CSC-associated drug resistance and recurrence, in vivo models and clinical trials are required.

Tumor development, invasion, and therapeutic resistance are all influenced by epigenetic processes, including histone acetylation. Numerous malignancies, including those of the lung, ovary, and mouth, have been found to overexpress the histone deacetylase HDAC6, which is linked to oncogenic transformation and increases proliferation, invasion, and migration [91,92]. Tavares et al. investigated the role of HDAC6 in chemoresistance and the CSC accumulation of oral carcinoma [93]. In tongue SCC cells CAL-27 (ATTC, CRL2095) and SCC-9 (ATCC, CRL-1629) that are CDDP-resistant, the authors [93] found elevated levels of HDAC6 as well as its nuclear accumulation. Additionally, CDDP-resistant cells had decreased ROS generation, low levels of DNA damage, and an increase of antioxidant protein PRDX2 [93]. Tavares et al. [93] looked at the possibility that inhibiting HDAC6 could reverse resistance with *Tubastatin A*, a pharmaceutical inhibitor of HDAC6. Remarkably, they had previously detected an accumulation of HDAC6 in their cell lines resistant to CDDP, and these cells exhibit increased susceptibility to HDAC6 inhibition. Moreover, CSCs showed greater susceptibility since tubastatin A at low concentrations effectively reduced this subpopulation, indicating that HDAC6 can be a viable therapeutic target to eradicate CSCs from OSCC.

Su et al. [94] demonstrated that *Fenofibrate* reduced the expression of markers for CSCs and their potential to spread, as well as the characteristics of cancer stemness. Furthermore, the authors [94] demonstrated that in CDDP Resistant (CR) OSCC-CSCs (derived from SAS, OECM-1, and GNM cell lines), fenofibrate reduced the expression of NF-κB p50 and p65 subunits. According to these findings, fenofibrate may be able to prevent tumor metastasis and recurrence. The authors [94] also showed that NF-κB downregulation might be a mediating factor for its anti-tumor actions. These findings demonstrated that fenofibrate can be used as an anti-CSC drug for OSCC and to enhance the efficacy of current treatments.

In young humans, *Dehydroepiandrosterone* (DHEA) is the most prevalent steroid in serum and serves as a precursor to sex hormones. As a dietary supplement, DHEA has been shown to have anti-inflammatory and anti-aging properties. DHEA has recently been researched in a variety of disorders, including cancer, utilizing medication repurposing techniques [95]. Several studies have demonstrated that DHEA affects cancer via various signaling pathways [96,97]. Li et al. examined how DHEA affected cancer stemness and HNSCC (derived from CAL 27, HSC-3, and SAS cell lines): the results showed that DHEA was less harmful to normal cells and had an inhibitory effect on HNSCC viability [95]. DHEA inhibits the WNT pathway in vitro and lowers tumorigenicity in vivo to produce anticancer effects, particularly with regard to the inhibitory effect of cancer stem-like cells [95]. These findings provide patients with HNSCC a fresh and encouraging treatment approach.

Roy et al. [98] affirmed that *XAV939* sensitized cells to CDDP and reduced the expression of stem cell markers (CD44, KLF4, OCT4, and β-catenin). According to this research, XAV-939 and CDDP combined therapy has the ability to be both cytotoxic and genotoxic by preventing CSC-mediated chemoresistance in HNSCC. Therefore, the combination treatment may have a positive effect on the course of treatment for HNSCC. To fully understand the result of this therapeutic approach, more research on a panel of chemoresistant cells and clinical trials of the combination are needed.

Also, Sinnung et al. also studied XAV939 [99]. The combination treatment of CDDP and XAV939 reversed the aberrant expression of β-catenin, caused CDDP sensitivity, and reduced CSC features on the 5–8F cell line. This research thus provides a novel chemotherapeutic that may decrease the characteristics of cancer stem cells while inhibiting the emergence of drug resistance in nasopharyngeal carcinoma.

Kumar et al. [100] selected *suberoylanilide hydroxamic acid* (SAHA), a pan-HDAC inhibitor for their study as it has been shown to demonstrate a potent antitumor activity against both hematologic and solid tumors [101]. For the first time, this research shows that SAHA therapy can reverse chemoresistance in CDDP-resistant cells by reducing nanog expression. Furthermore, by reducing the number of cancer stem cells, SAHA-mediated downregulation of nanog expression may also be preventing tumor spread. It was shown that SAHA considerably amplifies the antitumor effects of CDDP treatment for tumors that are both HPV-positive and HPV-negative on two head and neck cancer cell lines (one HPV-negative CAL-27 and one HPV positive UD-SCC-2) [101]. This is significant because it is predicted that soon, the majority of head and neck malignancies in the US will be HPV-positive [102]. For these individuals as well as those with HPV-negative HNSCC who are not responding to present treatment, targeting HDACs may be a helpful tactic.

Warrier et al. [103] have demonstrated the ability of *sFRP4*, an endogenously produced Wnt antagonist, to suppress CSC proliferation and increase these cells’ sensitivity to chemotherapy in the laryngeal cancer cell line Hep2 (Cellosaurus HEp-2, CVCL_1906) and tongue cancer cell line KB (Cellosaurus KB, CVCL_0372).

Since normal noncancerous cells have modest levels of Wnt signaling, sFRP4 is not inhibited in these cells. The results of this study should help sFRP4 become a more effective CSC inhibitor in a variety of tumor types.

It has been demonstrated that *curcumin-difluorinated* (CDF), a synthetic analog of curcumin, is more efficient than curcumin at inhibiting the proliferation of several human tumor cell lines [104,105]. The East Indian plant *Curcuma longa* is the source of *curcumin (diferuloylmethane)*, which is often used as a spice. It is regarded as pharmacologically safe and has been used for millennia as a dietary supplement. The low incidence of colon cancer in India is attributed by epidemiological research to the antioxidant and chemopreventive qualities of curcumin-rich diets. Although liposomal versions of curcumin have been employed in growth inhibitory studies of head and neck and pancreatic malignancies, curcumin is only soluble in organic solvents [106]. It has been demonstrated that curcumin-difluorinated (CDF), a synthetic analog of curcumin, is more efficient than curcumin at inhibiting the proliferation of several human tumor cell lines [104,105]. Curcumin and CDF, however, have never been demonstrated to directly prevent CSC development in HNSCC. Basak et al. [107] demonstrated the growth inhibition of curcumin and CDF on isolated and CSC-enriched CDDP-resistant head and neck cancer cell lines from CCL-23R and UM-SC-1R CDDP-resistant cell lines derived from Hep2 and CCL-23 cell lines, respectively. This implies that taking curcumin supplements could improve the way HNSCC’s CDDP treatment works. The authors think that a longer-term clinical trial of CDF using oral therapy for patients with head and neck cancer may yield information about the effectiveness of CDF in cancer therapies. It will also offer a chance to comprehend the long-term impact of CDF on cytokines and growth factors, as well as to assist in figuring out if CDF is harmful to cancer patients.

A branching short-chain fatty acid called *valproic acid (VPA)* increases chromatin acetylation by inhibiting histone deacetylases (HDACs) [108]. Numerous forms of human malignancies have been shown to overexpress or have mutations in HDACs [109]. According to a recent study, HNSCCs are predominantly hypoacetylated, which could explain how CSCs build up and remain in place [110]. Additionally, they demonstrated that clonogenic sphere formation was suppressed and the quantity of HNSCC CSCs was decreased by HDAC inhibition with Trichostatin A [110]. Regretfully, there are surprisingly few prior studies on the impact of VPA on HNSCC CSCs, so Lee et al. analyze how VPA affects HNSCC CSCs and identify the methods via which it impedes the traits of CSCs obtained from human primary HNSCC (K-3 and K-5 cell lines) [111]. They demonstrated that, when combined with CDDP, VPA may disrupt the CSC population in HNSCC, potentially offering a curative approach to the disease’s care [111].

As a prototypic inhibitor of the IL-6R signaling pathway, Herzog et al. employed *tocilizumab* (*Genentech*), a humanized monoclonal anti-IL-6R antibody that has been approved by the US Food and Drug Administration (FDA) for rheumatoid arthritis since 2010 [112]. These authors showed on HNSCC tissue microarray from humans (corroborated by in vitro results on UM-SCC-1 (Millipore Sigma #SCC070), -22A (Millipore Sigma #SCC076), and -22B (Millipore Sigma #SCC077) cell lines that Bmi-1 function is inhibited and that CDDP-induced CSC self-renewal and tumor growth are suppressed by therapeutic inhibition of IL-6R. Taken together, these findings imply that pharmacological suppression of IL-6R may represent a feasible approach to surmount CSC-mediated chemoresistance in head and neck cancer.

Nakano et al.’s work offers preliminary preclinical evidence (on cell lines UM-HNC.3b and UM-HNC-3A), for a novel approach to treating mucoepidermoid carcinoma (MEC), which combines platinum-based cytotoxic therapy (to debulk the tumor) with an mTOR inhibitor (to ablate CSCs) [113]. To maximize the effect of therapy on the survival of CSCs, Nakano et al. found that it is preferable to start treatment with the mTOR inhibitor and then administer CDDP, or to start the mTOR inhibitor and CDDP concurrently. The next steps will be to evaluate the effects of pretreatment with an immune checkpoint inhibitor (e.g., anti-PD-1 antibody) or an anti-stemness drug (e.g., small molecule inhibitor of Bmi-1) based on recent evidence from the Wang group in HNSCC [114].

**Table 2:** Molecules adjuvant to chemotherapy and their effects on CSCs.

	Mechanism of Action on CSCs	Effect on Conventional Chemotherapy
**ML-385** [91]	Inhibitor of NRF2; reduces CD44 expression	Increases effects of CDDP.
**Chloroquine (CQ)** [91]	Reduces CD44 expression	Increases effects of CDDP.
**Tubastatin A** [92,93,94]	Pharmaceutical inhibitor of HDAC6	Increases effects of CDDP.
**Fenofibrate** [95]	Reduces the expression of NF-κB p50 and p65 subunits.	Potential for increased effects of CDDP.
**Dehydroepiandrosterone (DHEA)** [96,97,98]	Inhibits the WNT pathway in vitro and lowers tumorigenicity in vivo	Unknown
**XAV939** [99,100]	Downregulates the expression of β-catenin	Unknown
**Suberoylanilide hydroxamic acid (SAHA)** [101,102,103]	Pan-HDAC inhibitor; reduces nanog expression in HPV-positive and -negative HNSCC	Increases effects of CDDP.
**sFRP4** [104]	WNT antagonist	Unknown
**Curcumin-difluorinated (CDF)** [105,106,107,108]	Synthetic analog of curcumin.	Unknown
**Valproic Acid (VPA)** [109,110,111,112]	Inhibits HDACs	Increases effects of CDDP.
**Tocilizumab** [113]	Humanized anti-IL-6R monoclonal antibody; inhibits Bmi-1 function.	Increases effects of CDDP.
**temsirolimus, BKM120, AZD8055, PF4708671** [114,115]	PI3k/mTOR signaling inhibitors; decreases Bmi-1 expression.	Increases effects of CDDP.
**Anti-PD-1** [115]	Decreases Bmi-1 expression	Increases effects of CDDP.

### 3.3. CSCs Targeting from Patient’s Fresh Biopsies for Functional Precision Therapy

Numerous studies in established head and neck cancer cell lines have been aimed at selectively combating cancer stem cells (CSCs). Recent studies using primary cancer cell cultures made from patient oral cancer biopsies showed that selectively enriched CSCs from the primary cancer cell cultures can be used in a chemosensitivity assay (ChemoID^®^) [27,28,115]. ChemoID^®^ is a proprietary assay for assessing drug responses, which has been designed to evaluate an individual’s malignant tumor response and facilitate the planning of a standard-of-care anticancer drug therapy determined by the treating physician [28,115]. Utilizing cutting-edge technology, ChemoID^®^ aims to identify chemotherapies capable of targeting and eliminating the CSCs, which serve as the foundation of cancer relapse [28,115]. This test quantifies the chemosensitivity and tumor response of an individual cancer patient to various chemotherapeutic drugs against both CSCs and the bulk of tumor cells. It provides valuable information on sensitivity and resistance, assisting oncologists in customizing the most effective chemotherapy combination tailored to the patient’s specific cancer cells. This approach translates into “individualized” chemotherapy based on the unique characteristics of the patient’s tumor, offering a strategic advantage against cancer [28,116,117]. ChemoID^®^ can be employed to select first-, second-, or third-line chemotherapies, irrespective of whether the goal of chemotherapy is cure or palliation. Additionally, the ChemoID^®^ assay is versatile, allowing it to be expanded to include in the screening process other new agents or other compounds/natural products aimed at chemosensitizing CSCs against conventional chemotherapies as already previously demonstrated [28].

As demonstrated in studies by Claudio P.P. et al. [17,27,28,29,30,31], the ChemoID^®^ drug sensitivity assay measures the survival of CSCs and the bulk of tumor cells cultured from human cancer biopsies following exposure to single chemotherapy drugs or their association. The ChemoID^®^ drug sensitivity assay has undergone testing and is presently used to assess the responsiveness of CSCs to various chemotherapy treatments in numerous solid malignancies, such as glioblastoma, ovarian, lung, breast, pancreatic, liver, and renal carcinoma, brain metastasis, metastatic colon carcinoma, and metastatic melanoma [27,34]. The ChemoID^®^ assay provides an advantage by assisting oncologists in individually selecting the most suitable chemotherapy regimen, particularly in situations where several equivalent options exist [28,118,119,120]. This assay enables the testing of various available chemotherapy drugs, including those considered standard-of-care, for their effectiveness against both CSCs and the overall mass of tumor cells (bulk cells). The ChemoID test can be particularly helpful in guiding the selection of chemotherapy in both cases of post-surgical cancer recurrence and non-operable tumors due to the poor health conditions of the patient [121]. In cases of substantial tumor recurrence after surgery, where a second surgery is not feasible due to the patient’s compromised health or the infiltration and extension of the tumor into vital structures, the use of a ChemoID^®^ assay to prevent the administration of resistant drugs becomes even more compelling. This is crucial because the diagnostic test has the potential to reduce unnecessary toxicity for patients who have already undergone unsuccessful chemotherapies and have a compromised performance status. The efficacy of this approach is also rooted in its safety, as the same biopsy procedure can be employed to obtain a second specimen for ChemoID^®^ purposes when performing a biopsy for histological diagnosis, thereby avoiding additional stress or risk for the patient.

In a recently published study by Spirito et al., the ChemoID^®^ assay was used to compare the chemosensitivity of 11 patients affected by advanced oral squamous cell carcinoma (OSCC) and found that there was a different response of each tumor to the chemotherapies tested by the assay, although the tumor histology was the same. The study also indicated that the sensitivity to chemotherapy did not appear to depend on the site of the squamous cell carcinoma [34]. The goal of the investigation was to verify if the chemosensitivity of OSCC cases was consistent with standard-of-care first-line chemotherapies indicated in the NCCN guidelines [122,123,124]. Although the effectiveness of chemotherapies recommended in current NCCN guidelines was confirmed in our study, it was shown that personalized therapy could be possible by screening chemotherapies before treating patients to discover more optimal treatments for each patient. These results highlight the potential of the ChemoID^®^ assay to individually target therapy for head and neck cancer patients, increasing the effectiveness of chemotherapy interventions. Treatments with expensive targeted anti-cancer drugs are not always feasible due to socio-economic and health disparity issues around the world.

## 4. Discussion

Natural compound-based therapies have been demonstrated to be very effective in treating cancer in vitro and more clinical studies are needed to demonstrate their value in adjuvant therapy to reduce side effects from conventional treatment and/or to increase the efficacy of anticancer treatments. Several botanical extracts (mainly from plants) that have an adjuvant role to first-line chemotherapies (mainly CDDP) in HNSCC have been studied in preclinical investigations using established human cancer cell lines and immune-deficient mice.

One of the limitations of our systematic review is that the majority of the published studies we analyzed were conducted on established cancer cell lines derived from oral cancer that were cultured for several passages, which do not take into consideration the phenotypic differences between HPV-positive and HPV-negative cancers or the sex-based differences in treatment response. This is important since CSCs in HPV-positive HNSCC are less capable of repopulation during therapy, presenting better treatment compliance than their HPV-negative counterparts [125,126]. However, we cannot fully discuss this since most of the in vitro studies described in our review were not undertaken on cell lines accounting for HPV status or both sexes.

CSCs have a paramount role in cancer progression, metastases, and chemoresistance. Most of the studies on the sensitivity of CSCs to chemotherapy have been conducted on commercial CSC lines, where stem properties may be modified after several passages of cell culture. ChemoID is the only diagnostic assay that measures the chemosensitivity of individual patients’ CSCs primary line against single chemotherapy drugs and their associations, thus providing patient-specific therapeutic indications.

Moreover, equivalent drug concentration used against CSCs culture in the ChemoID assay plays an important role in clinical therapy. ChemoID^®^, by using equivalent drug concentration inside the clinical range of toxicity, stands as the first drug response assay accessible to the clinic, to examine CSCs from solid tumors. Findings from the study conducted by Howard et al. [30] propose that a drug response assay targeting CSCs could prove highly beneficial in refining treatment selection, especially in cases of first-line therapy failure and when there are multiple clinically acceptable and equivalent treatment options available. Additionally, the results imply that personalized functional drug response assays might offer a broader array of treatment choices, leading to improved outcomes for a more extensive patient population compared to the current ex post studies on empirical, population-based treatment approaches. These compelling findings also indicate the potential reasonability of proactively employing functional testing with drug response assays like ChemoID^®^ to aid clinicians in optimally prioritizing therapy for glioblastoma multiforme (GBM) patients. In a randomized trial (NCT03632135), the ChemoID^®^ assay improved the median overall survival (mOS) of patients with recurrent GBM over physician-chosen chemotherapy [32]. The trial examined the utility of personalized chemotherapy based on the ChemoID^®^ assay results over chemotherapy regimens chosen by physicians. In the ChemoID assay-guided group, median survival was 12.5 months (95% confidence interval [CI], 10.2–14.7) compared with 9 months (95% CI, 4.2–13.8) in the physician-choice group (*p* = 0.010). Based on this data, the ChemoID^®^ assay recently received Breakthrough Device Designation status from the Food and Drug Administration (FDA) for the treatment of GBM.

The ability of the ChemoID^®^ assay to personalize chemotherapy selection is a promising way to provide more affordable treatment for head and neck cancer patients who may need chemotherapy treatments. The potential future application of the ChemoID assay could involve treating extensive cancer lesions in the maxillofacial area through a combination of surgery and targeted chemotherapy against cancer stem cells (CSCs). In such instances, the ChemoID assay could be employed to further assess the sensitivity of CSCs from a relapsed tumor against a broader range of drugs. Also, this test could prove more beneficial if incorporated as a routine examination in earlier stages, especially in situations where there is a need to substitute the surgical therapeutic approach with chemotherapy. However, more studies involving head and neck tumors will be necessary to acquire necessary objective data regarding the effectiveness of drugs on different types of head and neck tumors. This is essential to prevent unnecessary toxicity for patients and reduce costs for the healthcare system. The selective and specific analysis of chemosensitivity provided by a test like the ChemoID^®^ assay will prove value in the development of innovative therapies and the evaluation of new chemotherapy drugs for the clinical management of head and neck cancer.

## 5. Conclusions

CSCs have a paramount role in cancer progression, metastases, and chemoresistance. Most of the studies on the sensitivity of CSCs to chemotherapy have been conducted on commercial CSC lines, where stem properties may be modified after several passages of cell culture. ChemoID is the only diagnostic assay that measures the chemosensitivity of individual patients’ CSCs against single chemotherapy drugs and their associations, thus providing patient-specific therapeutic indications. The role of the ChemoID assay in cancer therapy has been supported by phase-3 clinical trial studies in recurrent glioblastoma and recurrent platinum-resistant ovarian cancer, which have supported a Breakthrough Device Designation by the FDA for its clinical use in glioblastoma.

The potential future application of the ChemoID assay could involve treating extensive cancer lesions in the maxillofacial area through a combination of surgery and targeted chemotherapy against cancer stem cells (CSCs).

## Abbreviations

Oral Squamous Cell Carcinoma (OSCC); Head and Neck Cancer (HNC); Head and Neck Squamous Cell Carcinoma (HNSCC); Cancer Stem Cells (CSCs), cisplatin (CDDP).
